# Deterrence approach on the compliance with electronic medical records privacy policy: the moderating role of computer monitoring

**DOI:** 10.1186/s12911-019-0957-y

**Published:** 2019-12-04

**Authors:** Kuang-Ming Kuo, Paul C. Talley, Tain-Junn Cheng

**Affiliations:** 1 Department of Healthcare Administration, I-Shou University, No.8, Yida Rd., Yanchao District, Kaohsiung City, 82445 Taiwan, Republic of China; 2 Department of Applied English, I-Shou University, No. 1, Sec. 1, Syuecheng Rd., Dashu District, Kaohsiung City, 84001 Taiwan, Republic of China; 3 Departments of Neurology, and Occupational Medicine, Chi Mei Medical Center, Tainan, Taiwan, Republic of China; 4 Department of Health Management Center, Chi Mei Medical Center, Taiwan, Taiwan, Republic of China

**Keywords:** Electronic medical records, Privacy policy, Sanction certainty, Compliance intention, Deterrence theory, Regulatory compliance

## Abstract

**Background:**

This study explored the possible antecedents that will motivate hospital employees’ compliance with privacy policy related to electronic medical records (EMR) from a deterrence perspective. Further, we also investigated the moderating effect of computer monitoring on relationships among the antecedents and the level of hospital employees’ compliance intention.

**Methods:**

Data was collected from a large Taiwanese medical center using survey methodology. A total of 303 responses was analyzed via hierarchical regression analysis.

**Results:**

The results revealed that sanction severity and sanction certainty significantly predict hospital employees’ compliance intention, respectively. Further, our study found external computer monitoring significantly moderates the relationship between sanction certainty and compliance intention.

**Conclusions:**

Based on our findings, the study suggests that healthcare facilities should take proactive countermeasures, such as computer monitoring, to better protect the privacy of EMR in addition to stated privacy policy. However, the extent of computer monitoring should be kept to minimum requirements as stated by relevant regulations.

## Background

Hospitals have become increasingly aware that electronic medical records (EMR) have the potential to provide many benefits, such as improved healthcare quality, reduced medical errors, decreased costs [1], and professional staff access to patient information without limitations of either time or space [1]. EMR have also been well recognized as a cost-effective investment to make [2–4]. More and more healthcare facilities have thus adopted EMR to maximize benefit from the eventual trend of digitalization.

However, an increasing reliance on EMR has led to a corresponding increase in the possible negative influences risked in EMR breaches from unauthorized access to EMR by internal staff or outside sources. These breaches may cause intangible/tangible damage to both hospitals and private individuals alike [5] since the burgeoning volume of digital medical records remains highly accessible to both authorized and unauthorized users [6]. According to a U.S. Health and Human Services Department report [7], there have been 329 reported breaches involving an incident in which more than 500 records were exposed. More specifically, there were a total of 16,471,765 patients whose medical records were breached intentionally or unintentionally in 2016 alone. Most of these reported incidents of privacy violations in healthcare facilities stem in fact from staff misuse or abuse of their privileged access relationship to patient records [7, 8]. What is more important is that if the information is disclosed inappropriately, patients may receive serious harm [6]. It should be widely understood that non-compliance with the privacy rules may encompass both civil lawsuits and criminal penalties in many countries [9–11]. In Taiwan, for example, the maximum civil monetary penalty can be more than six million USD, accompanied by five years of imprisonment, if related privacy-protection regulations are found to be broken.

In the realm of information security, literature [12] has suggested four security activities to ameliorate the problem of unauthorized access, namely, an implementation of deterrence, prevention, detection, and remedies which will reduce the considerable number of inherent security risks. Deterrence refers to how organizations can best deter a potential perpetrator from committing unlawful behaviors by indicating serious sanctions related to security breaches, and that organizations will certainly punish breaches heavily through these proscribed rules (i.e., organizational policies) [13]. Prevention refers to the use of active countermeasures (e.g., physical locks of information assets or password protection) with ready abilities that will prevent illegitimate intentions and unauthorized intrusions. Detection, such as computer monitoring, provides for the purposeful investigation of activities in order to identify plausible abnormalities. Remedies refer to whatever an organization can do to recover from the harmful effects of security-violation issues [12]. Among these four activities, deterrence and detection exist as well-established influences to dissuade employees’ unlawful/unauthorized behaviors. These behaviors include the violation of organizational policies or the compelling employees’ compliance intentions [13–18].

The deterrence theory, which mainly states that individuals are less likely to undertake illegal behaviors if the pertinent sanctions are severe and certain, is one of the many theories [14, 16, 19–21] that have been widely adopted to investigate compliance to security policy. Extant literature however often reported mixed results [22] when utilizing the deterrence theory for modeling compliance to information security policy. To better understand the plausible effects of deterrence such as sanction severity/certainty, prior study has called for testing more contingency variables and their possible moderating effects [22]. Further, literature [23, 24] suggests that the identification of moderating effects is important to advance scientific knowledge in the field. However, the moderating influence of detection practices on the relationship between deterrence and policy compliance intention is seemingly less investigated. We therefore contend that an identification of the moderating effects of detection practices could elicit differing perspectives as to the furtherance of organizational-policy compliance studies.

The primary purposes of this quantitative research were two-fold: 1) to investigate the inherent relationships between deterrent practices (i.e., sanction severity and sanction certainty in our study) and EMR privacy policy compliance intentions among hospital employees; and, 2) to explore the moderating influence of detection practices (i.e., computer monitoring in our study) on those relationships, as stated above. The results of our study should be of interest to both academics and practitioners pertinent to healthcare industries.

## Theoretical background

### Computer monitoring

The use of monitoring oversight in workplaces to protect business information/assets, to encourage productivity, and to evaluate performance has significantly increased over the years [25]. It has been greatly facilitated through technological advancements [26]. Among the various monitoring technologies currently available, computer monitoring, referring to the use of computer technology to automatically collect work-related information, such as the tracking employees’ Internet usage, recording network activity, and performing security audits [26], has been regarded as an effective means to gain an employee’s compliance with stated organizational rules or policies [27].

In their study of employees’ reactions to forms of monitoring, Chang et al. [27] found that a perceptible amount of monitoring serves to lower employees’ trust in an organizations’ purpose, despite the retention of such trust being able to improve employees’ compliance with such organizational monitoring. However, other studies reported that computer monitoring can exert a negative impact on employees’ attitudes or even upon their compliance intentions due to its perception as being intrusive. For example, Jeske and Santuzzi [28] reported that the psychological influences of electronic performance monitoring include negative job attitudes and reduced self-efficacy. Further, Spitzmüller and Stanton [29] reported that employees’ attitudes toward surveillance and monitoring are significantly and negatively associated with their compliance intentions regarding oversight. Finally, the monitoring of employees has also been reported to be of mixed acceptance [26]. In view of the alternately good and bad results of evident computer monitoring effects, further studies are suggested to afford a better understanding of this phenomenon and compliance-related issues.

### Deterrence theory

Originating from the criminology discipline, deterrence theory assumes that individuals will make rational decisions regarding the commission to commit crime based on a trade-off between the benefits and costs of that commission [22]. When the benefits are said to outweigh the costs, individuals may choose to undertake illegal behavior [30]. Consequently, deterrence theory states that individuals’ unlawful behaviors can be deterred via severe, certain, and swift legal sanctions that lead to certain costs [31, 32]. Deterrence theory comprises three major constructs, namely sanction severity, sanction certainty, and sanction celerity [22, 33]. Sanction severity refers to the degree of punishment relative to the illegal acts [31]. Intuitively, the more severe a sanction may become, a rational individual will choose not to take such illegal acts. Furthermore, certainty of sanctions means a punishment that is certain to occur whenever an unlawful behavior has been committed. Therefore, if punishment is assured, individuals will be also become dissuaded from such illegal behaviors [33, 34]. Finally, sanction celerity means the extent to which sanction is swift in order to affect deterrence of a crime [33]. Among the aforementioned constructs given over from deterrence theory, sanction severity and sanction certainty are the two most investigated constructs, and sanction celerity is rarely included in the literature [22, 33]. In their meta-analysis of deterrence theory, Pratt et al. [30] found that the effect size of sanction certainty is more substantively important than that of sanction severity especially when predicting ‘white-collar’ types of offenses such as fraud, tax violation, non-compliance with regulatory code. Sanction celerity was not considered as a possible integer.

In recent years, a considerable amount of literature has adopted deterrence theory within the context of organizational policy compliance/violation investigation. More specifically, many studies used the constructs connected to deterrence theory in order to explain the intention of information security behaviors, such as the following: information security policy compliance intention [15, 16, 35, 36]; intention to violate information security policy [37, 38]; information systems misuse intention [13, 14, 17, 39, 40]; internet use policy compliance [41]; and, information systems security effectiveness [42].

Prior literature [43, 44] classified these deterrence constructs into three categories: 1) Security policy, 2) security awareness, and 3) security systems. A security policy is used to define employees’ roles and responsibilities regarding information security by stated policies. Security awareness aims to inform employees about the importance of security and the consequences of security threats [12]. Both security policy and security awareness are considered as passive countermeasures to information security threats [13]. Finally, security systems are an active countermeasure used to enforce security policies by means of detection system activities executed with the assistance of computer applications [12, 13]. Appendix Table A1 shows the selected literature that has adopted deterrence theory to explain information security-related issues. Despite the strong theoretical foundation in criminology [30], the studies that have adopted deterrence theory have reported mixed results in an information security context. D’Arcy and Herath [22] argued that such inconsistencies can be resolved in the following ways: 1) Identifying contingency variables, 2) evaluating methodological issues, and 3) conducting better substantive research question reviews.

#### Sanction severity

In our study, sanction severity refers to the degree of punishment pertinent to non-adherence to stated EMR privacy policy [14]. In terms of sanction severity, the deterrence theory [31, 32] suggests that if the level of sanction increases, an individual will be less likely to act illegally. In information security research, several studies [13–15, 17, 18] found that, via severe punishment, employees are more likely to comply with organizational security policies, or are less likely to misuse information systems. In our research context, if the level of sanction increases conversely, hospital employees are more likely to adhere to stated privacy policy as a result. Otherwise, they are subject to punishment with severe civil or criminal penalties if they are caught breaking stated privacy policy. Hence, we anticipate the following:

H_1_: Sanction severity has a positive relationship on hospital employees’ intentions to comply with EMR privacy policy.

#### Sanction certainty

Not only sanction severity but also sanction certainty are known to regulate individuals’ behaviors [31, 32]. In our study, sanction certainty means the real probability of receiving punishment related to non-adherence to EMR privacy policy [14]. The deterrence theory presumes that potential perpetrators are made aware of compliance assurance efforts such as rules and punishments necessary to restraint of illicit behaviors [15]. In an organizational context, rules for regulating employees, however, will not be effective if the rules are not immediately enforceable [45]. Therefore, if employees’ misbehaviors are circumvented, and they become fully aware that they will undoubtedly be penalized for such misbehaviors, employees will then more likely comply with stated rules and regulations. Existing studies of information security also lend support to this notion [13, 17]. Transferring this rationale to our study, if hospital employees become aware that the probability of their being punished is certain whenever violating stated privacy policy, there is greater likelihood that they will abide by the stated privacy policy and avoid such transgression. We therefore propose:

H_2_: Sanction certainty has a positive relationship on hospital employees’ intention to comply with EMR privacy policy.

#### Computer monitoring in the workplace for policy compliance

In addition to the oft-repeated claim that perceived severity of, and direct certainty of, sanction have a mitigating influence on an individual’s deviant behavior [14], deterrence studies also indicate conversely that monitoring and surveillance have the potential to reinforce perceived severity of, and perceived certainty of, sanction [14, 17, 39]. In their review of deterrence theory, D’Arcy and Herath [22] further discussed several contextual factors which may moderate the relationships between the major components of deterrence theory and policy compliance intention. One of these contextual factors is “virtual status” which refers to the degree employees perform work remotely [22]. Results indicate that the deterrence effects of monitoring on remote workers is far weaker than on central workers because they are less monitored [39].

Since deterrence (i.e., sanction severity and sanction certainty in our study) and detection (i.e., computer monitoring in our study) have already been well-studied to predict individuals’ unlawful behaviors [14–18], taken from the perspective that detection can strengthen deterrence effect [12], we argue that the associations between the sanction severity/sanction certainty of deterrence theory and policy compliance intention are contingent on the level of computer monitoring performed. This finding is seldom investigated in prior studies. Therefore we only investigate computer monitoring as a moderator instead of as an antecedent of compliance intention.

Under a higher level of computer monitoring, employees are less likely to violate organizational policy due to the real probability of being caught; therefore, employees will comply with stated policy no matter what the implied severity statement is or certainty of sanction may be. It is thus reasonable to expect that the association between sanction severity/certainty and policy compliance intention will be lower for employees who have a perceived higher level of computer monitoring. On the other hand, employees who perceive a lower level of computer monitoring will become more sensitive to the magnitude of effect that sanction severity or sanction certainty has on their intention to comply with stated policy. Those employees may hold that the chance of being caught for violating stated policy is considerably lowered because they are less computer-monitored. However, if they are aware of the severity of and certainty of sanction, they are more likely to also perceive the obvious effects of deterrence (i.e., sanction severity and sanction certainty) that are in place [39]. According to the above discussions, the following hypotheses are then proposed:

H_3_: Compared with hospital employees who have a lower perception of computer monitoring, the relationship between perceived sanction severity and their intention to comply is stronger than that of hospital employees with a higher perception of computer monitoring.

H_4_: Compared with hospital employees who have a lower perception of computer monitoring, the relationship between perceived sanction certainty and their intention to comply is stronger than that of hospital employees with a higher perception of computer monitoring.

**Fig. 1:**
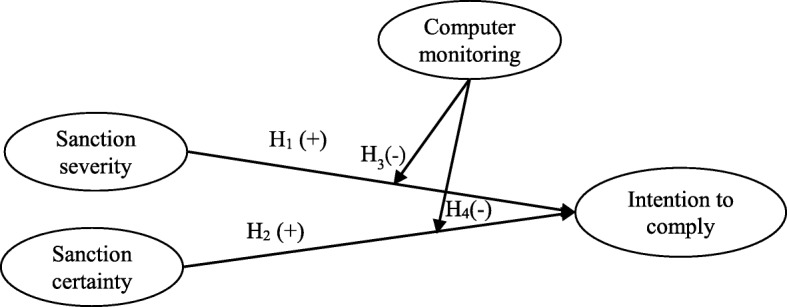
Research model

The research model for this study is depicted in Fig. 1. While the research model may appear simple, it may yet provide additional knowledge about the compliance construct. Compliance (to privacy policy), being a complex construct [21], and its relationships with other constructs, are intricate and thus require considerable investigation. By focusing on a much smaller part of the complex relationship in store, we may start to probe and to understand just how such a complex phenomenon may take place.

## Methods

### Measures

The instrument used in the present study consisted of two parts. The first part deals with the demographic data of respondents, and the second part ascertains respondents’ perceptions related to sanction severity, sanction certainty, computer monitoring, and their intention to adhere to stated EMR privacy policy. These research constructs were assessed using validated instruments containing sufficient reliability and validity [14, 15, 38, 46, 47]. Sanction severity, adapted from [15], was measured by two items indicating the degree of punishment pertinent to non-adherence to stated EMR privacy policy. One example item for sanction severity was: “My hospital disciplines employees who break EMR privacy rules.” Sanction certainty, measuring the real probability of receiving punishment relating to non-adherence of EMR privacy policy, was adapted from [38, 46] and included three items. One example question for sanction certainty was: “If I don’t follow EMR privacy policies, I will be penalized.” Three items were rated to measure computer monitoring, referring to the likelihood of detection non-adherence of EMR privacy policy, and was adapted from [14]. One example item for computer monitoring was: “I believe that my hospital monitors any modification or altering of EMR by employees.” Intention to comply with EMR privacy policy was adapted from [47] and was measured by use of three items. One example question for intention to comply with EMR privacy policy was: “I intend to continue complying with EMR privacy policy in the future.”

Except for demographic questions, all survey questions utilized a 7-point Likert scale (e.g., 1 = *strongly disagree*, and 7 = *strongly agree*). Since the original adopted items were given in English, we were obliged to translate these items into Chinese for purposes of administration. The back-translation [48] approach was adopted to ensure that the meaning of the original items was preserved during the translation between Chinese and English. We conducted a pilot test to construct the scales via 30 healthcare professionals located in a large medical center. Modification of wording was made to items resulting in a final scale which was justified for further testing (*see* Appendix Table A2).

### Participants

The subject hospital has nearly 1200 beds and attracts an average of nearly 5000 outpatients each day, and it has adopted EMR methods since 2010. Having a total of 3511 employees, including 3020 healthcare professionals and 491 administrative staff, about 2800 healthcare professionals and 100 administrative staff of the subject hospital were authorized to access EMR. Those privileged EMR users are mandated to take various EMR-related training programs, such as medical ethics, personal information protection, or disaster recovery of EMR systems, indicating that they are now qualified to participate in this study. Considering the heavy workload of many hospital employees, a census of all eligible employees is as yet unfeasible, we therefore adopted convenience sampling to collect relevant data pertaining to this study. We appointed a coordinator for the clinical and administrative departments whose staff members have access to EMR systems to assist with the dissemination and collection of the questionnaires. Among the 2900 eligible hospital employees from differing units, we distributed 350 questionnaires to those units that were willing to participate in our survey. Permission from the Institutional Review Board of a medical center was obtained prior to investigation. From February to April in 2015, 310 voluntary and anonymous responses were collected, indicating a response rate of 88.57%. Excluding seven incomplete responses, we were left 303 responses for later analysis.

## Results

### Demographic profile of respondents

Of the 303 valid responses given, 60.07% of respondents were female. Approximately 77.88% of the respondents were 30–49 years of age. Further, most respondents were college- or university-educated (78.55%). Over 71.61% of respondents have more than 5 years of working experience in the healthcare industry, indicating they should have sufficient knowledge for inclusion in our study. Details of the participants are depicted in Table 1.

**Table 1:** Descriptive statistics of respondents’ characteristics

Characteristics	Items	Frequency (n)	Percentage (%)
Gender	Male	121	39.93
	Female	182	60.07
Age	20–29	43	14.19
	30–39	132	43.56
	40–49	104	34.32
	> = 50	24	7.92
Education	High school	3	0.99
	College	18	5.94
	University	220	72.61
	Graduate school	62	20.46
Title	Nurse	33	10.89
	Physician	100	33.00
	Other healthcare professionals	69	22.77
	Administrative staff	101	33.33
Experiences in healthcare industry (years)	1–5	86	28.38
	6–10	66	21.78
	11–15	46	15.18
	16–20	72	23.76
	> = 21	33	10.89

Note: Some of the total percentage may be over/under 100% due to rounding off

### Reliability and validity test

We used Cronbach’s alpha (α) and principal components analysis (PCA) to assess construct reliability and construct validity in our study. Further, PCA is useful to define the underlying structure among the measurable variables (items) contained in the analysis. As depicted in Table 2, the Cronbach’s alpha values range from 0.85–0.95, indicating sufficient reliability [49]. Further, the Kaiser-Meyer-Olkin (KMO) measure verifies the sampling adequacy with KMO = 0.91 [49]. Bartlett’s test of sphericity, χ^2^(66) = 3268.87, *p* < .001, demonstrating correlations of items, is sufficient for purposes of PCA [49]. With varimax rotation, four factors with eigenvalues of at least one were extracted. Convergent validity can be confirmed if the items load highly on their respective factors, while discriminant validity can be verified if each item loads higher on its posited factors, rather than on other factors [49]. Table 2 further demonstrates that all items have factor loadings > 0.55 on their posited factors and load highly on the posited factors, rather than on alternate ones. Reliability and construct validity are thus determined to be adequate for purposes of our study parameters.

**Table 2:** The results of factor analysis

Variables	ITC	CM	SS	SC
SS1	0.23	0.24	**0.72**	0.27
SS2	0.31	0.23	**0.76**	0.33
SS3	0.20	0.20	**0.81**	0.18
SC1	0.15	0.19	0.38	**0.80**
SC2	0.37	0.22	0.26	**0.78**
SC3	0.52	0.29	0.22	**0.55**
CM1	0.32	**0.84**	0.22	0.18
CM2	0.29	**0.86**	0.25	0.17
CM3	0.29	**0.79**	0.21	0.22
ITC1	**0.77**	0.37	0.29	0.23
ITC2	**0.81**	0.34	0.30	0.25
ITC3	**0.79**	0.34	0.26	0.26
Eigenvalue	2.79	2.76	2.42	2.06
Variance Explained (%)	23	23	20	17
Cronbach’s Alpha	0.95	0.93	0.85	0.85

Note: SS = sanction severity, SC = sanction certainty, CM = computer monitoring, ITC intention to comply

Boldface, factor loading structure

After identifying the structure between the scale items and the four constructs investigated in our study, the scores of these constructs were calculated by averaging the scores of their corresponding items. Table 3 depicts means, standard deviations, and correlation coefficients for all constructs at hand. Since one correlation coefficient is larger than 0.7, we further examined for the collinearity issue. The results demonstrated that the tolerance value of sanction severity, sanction certainty, and computer monitoring ranges from 0.41–0.56, revealing that collinearity should not be seen as a problem in our study [49]. D’Arcy and Herath [22] argued that there is potential overlap among the measures of deterrence constructs which may contribute to higher correlation coefficients.

**Table 3:** Means, standard deviation, and inter-correlations

	*M*	*SD*	SS	SC	CM	ITC
Sanction severity (SS)	5.67	0.84	–			
Sanction certainty (SC)	5.59	0.80	0.71**	–		
Computer monitoring (CM)	5.26	0.83	0.60**	0.61**	–	
Intention to comply (ITC)	5.51	0.83	0.55**	0.59**	0.69**	–

Note: ** *p* < 0.01

### Testing of hypotheses

Hierarchical regression analysis, adopted by many studies for discovering moderating effects [50–52], was used to test the study’s hypotheses. The ratio of observations for each independent variable in our study was higher than the suggested 20:1 necessary for the conduct of multiple regression, indicating the results should be generalizable if the sample is at once representative [49]. However, we had to adopt convenience sampling to collect eligible respondents since we could not enforce all eligible hospital employees to take part in our survey, which may lower the generalizability of our findings.

We followed advice provided by Hair et al. [49] used to determine whether the moderator effect is significant within a three-step process: 1) to estimate the un-moderated model; 2) to estimate the moderated model (i.e., to include the interaction terms); and, 3) to assess the statistical significance of the additional variance explained by the moderator. As per the validating process, two models were estimated. Model 1 assessed the relationship between independent variables (i.e., sanction severity and sanction certainty) and the moderating variable (i.e., computer monitoring) on the dependent variable (i.e., intention to comply) found in this study. The results revealed that sanction severity (β = 0.159, *p* = .002), sanction certainty (β = 0.361, *p* < .001) and computer monitoring (β = 0.410, *p* < .001) were all significant, providing support for H_1_ and H_2_. Model 2 builds on Model 1 but includes two interaction terms, namely sanction severity*computer monitoring and sanction certainty*computer monitoring. To reduce multi-collinearity, all three variables were first mean-centered before being multiplied by each other [53]. A partial *F* test (*see* Table 4) demonstrated that Model 2 explained significantly more variation than Model 1 [∆*R*^*2*^ = 0.01, *F* (2, 297) = 4.533, *p* = .011]. The interaction of sanction severity and computer monitoring was not significantly associated with intention to comply. Model 2 however provides evidence to suggest that computer monitoring moderates the relationship between sanction certainty and one’s intention to comply (β = − 0.138, *p* = .008). The results of hierarchical regression tests thus provide support of H_4,_ but not of H_3_.

**Table 4:** Regression analysis of the effect of sanction severity/certainty on the intention to comply

	Model 1		Model 2	
	Standardized β	Tolerance	Standardized β	Tolerance
Independent variable				
SS	0.159**	0.456	0.164**	0.412
SC	0.361**	0.444	0.381***	0.436
Moderating variable				
CM	0.410***	0.571	0.425***	0.563
Interactions				
SS*CM			0.048	0.382
SC*CM			−0.138**	0.407
*R*^*2*^	0.663		0.673	
Adjusted *R*^*2*^	0.660		0.668	
*F* value	196.452***		122.493***	
∆*R*^*2*^			0.01	
*F* value for ∆*R*^*2*^			4.553**	
*df*	299		297	

SS = sanction severity, SC = sanction certainty, CM = computer monitoring

**p < .01, *** p < .001

**Fig. 2:**
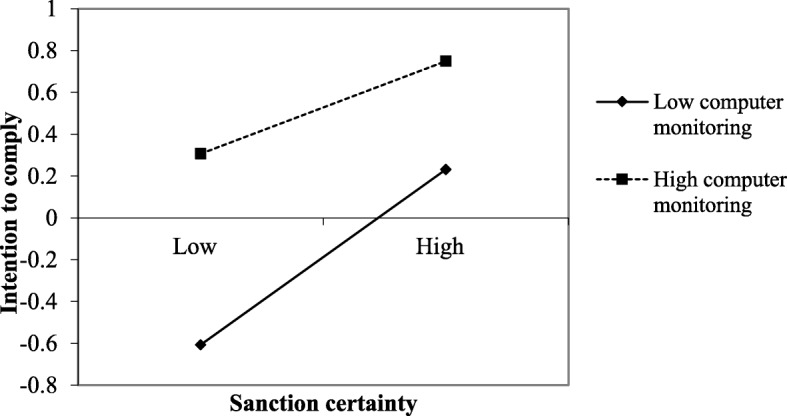
Relationship of sanction severity and intention to comply for two levels of computer monitoring

To better understand the influence pattern of the interaction between sanction certainty and computer monitoring, we plotted the interaction graphically as suggested by Aiken and West [53]. Figure 2 depicts the relationship between sanction certainty and intention to comply at two levels of computer monitoring. The figure demonstrates that the relationship between sanction certainty and intention to comply was stronger among hospital employees who perceived a lower level of computer monitoring taking place versus among hospital employees who perceived a higher level of computer monitoring.

### Subgroup analysis of non-significant moderating effects

Since computer monitoring did not demonstrate a significant moderating effect on the relationship between sanction severity and intention to comply (H_3_), we conducted a less conservative subgroup analysis [54]. The total sample was divided into low (*n* = 164) and high (*n* = 139) perception of computer monitoring groups according to the established median. We then assessed the moderating effect of computer monitoring by comparing correlation coefficients according to the suggestion made by Arnold [55]. Specifically, the correlation coefficient between sanction severity and intention to comply in the high-perception-of-computer-monitoring group (*r* = 0.590, *p* < .01) was higher than what was observed in the low-perception-of-computer-monitoring group (*r* = 0.397, *p* < .01). The *t*-test provides evidence that the two correlation coefficients are significantly different (*z* = − 2.21, *p* = .027). We thus claim partial support for H_3_.

## Discussion

As previously highlighted, the protection of electronic medical records privacy is an important managerial issue given its extensive proliferation among healthcare facilities and the extent to which EMR can change the paradigm of the healthcare service provided. An effective hospital staff adherence to stated privacy policy will enable patients to have more trust in the services delivered, and hospital employees can confidently access patient-related information instantly, regardless of time and location. The positive intention of compliance coupled with stated privacy policy will thus tend to improve the overall quality of healthcare service, mitigate the risks and legal consequences that healthcare facilities might face, and lower the potential negative impact on patients through possible security breaches to EMR.

Based upon this understanding, the main goal of our study has been to examine theoretical factors that may improve hospital employees’ intention to adhere to stated privacy policy of EMR from a deterrence perspective. To this end, our study has highlighted two conceptual realms: 1) deterrents such as sanction severity and sanction certainty towards compliance intention, and 2) the effects of computer monitoring on the effects of such available deterrents.

The main finding of our study is a determination of the moderating effect that computer monitoring has upon sanction severity and sanction certainty towards hospital employees’ adherence to stated privacy policy. Even though the literature [23, 24] suggests that an identification of moderating effects is important towards the advancement of scientific knowledge, relatively few studies have tested the moderating effects of computer monitoring on existing associations between deterrents and compliance intentions. The results of the moderating effects in our study showed that the association between sanction certainty and behavioral intention was stronger among hospital employees with a lower-level-of-computer monitoring. That is, with low levels of computer monitoring, it is particularly true that if hospital employees know of the certainty of sanctions that they will inevitably adhere to stated privacy policy. This finding is in accordance with the study by D’Arcy and Hovav [39], who found that the deterrence effects of monitoring on remote-site workers are weaker than central workers because they are in fact less monitored. In other words, remote-site workers may thus not behave as accordingly as centralized workers may do. Literature [14] has encouraged the practice of computer monitoring because it is an effective countermeasure for regulating inappropriate information security behaviors; and, most importantly, organizations can directly control such a mechanism on a regular basis. Based on the findings of the moderating effect of computer monitoring, we, however, suggest that healthcare facilities should continue to monitor the usage of EMR, but employees should not be negatively influenced by or come to suspect such surveillance activities routinely take place. This suspicion may lower the performance of employees due to invisible pressures [28, 29]. Most importantly, healthcare facilities should make sure that their employees are aware of the computer monitoring that is taking place, and the severity of and certainty of sanctions whenever stated privacy policy is violated. This is especially true since deterrence effect may be maximized if potential perpetrators are fully aware of the certain consequences of illegal behaviors [12].

Besides, consistent with previous studies [13, 14, 17, 18], sanction severity and sanction certainty were significantly related to one’s intention to comply with stated privacy policy. This may imply that both sanction severity and sanction certainty are effective determinants for regulating hospital employees’ future policy-compliance behavior. In terms of the relative importance of these two determinants, sanction certainty demonstrated a stronger predictive measure than sanction severity, which corroborates with the findings of meta-analysis by Pratt et al. [30]. Pratt et al. [30] also argue that sanction certainty tends to perform the best when predicting “white-collar” types of offenses, which is consistent with our study. According to the findings, we suggest that healthcare facilities should clearly define a set of policies with detailed rules and regulations regarding the potential punishments for all unlawful behaviors involving EMR. And most importantly, these policies should be communicated to hospital employees via training sessions. By doing so, potential offenders are more likely to be dissuaded from committing unlawful behaviors by the possibility of incrimination.

Our study contributes to both academic and practical concerns related to EMR administration. From an academic standpoint, our study provides one of the few tests of the differential deterrence hypothesis in the realm of EMR privacy protection. With few exceptions [14, 39], most studies from the IS security have presumed that the impact of deterrents is consistent across most given individuals. By investigating the moderating effect of computer monitoring, our study contributes to a better understanding of the relationships between deterrents (i.e., sanction severity/certainty in our study) and policy compliance intention.

From the perspective of EMR privacy protection, the results may demonstrate that the effectiveness of sanction certainty is reliant upon hospital employees’ perceived levels of computer monitoring. The higher level of computer monitoring perceived by hospital employees, the lower the effect of sanction certainty on compliance intention will be. Therefore, healthcare facilities should inform their employees that EMR usage and access are duly monitored according to the security requirements and privacy concerns deemed necessary by the healthcare authorities. No excessive monitoring practices are implemented in their healthcare facilities. Further, any monitoring must be carried out in the least intrusive way possible. This is especially important as more health facilities have commonly adopted EMR practices for most procedures, to the extent that many hospital employees can now only acquire and maintain patients’ medical records from EMR systems.

Like most empirical studies, our study has limitations that should be taken into account. First, the study sample is drawn from only one medical center in Taiwan. Therefore, inferences to the wider population may not be safely made. In other words, the external validity of the present findings may therefore be limited to a greater or lesser extent. Since we adopted a convenience sampling approach, the participants may not be representative of all eligible hospital employees. Our findings can only become generalized to a population with the same characteristics. Further, the survey was mainly based on self-report rather than direct observation or the monitoring of participants’ regular behavioral patterns. Future research can thus examine the issue in order to better understand the associations among these constructs. Further, since our questionnaires asked about hospital employees’ intention to comply with EMR privacy policy, they may tend to behave in a rule-obedient manner despite the survey being completely voluntary and anonymous. Hence, the possibility of social acceptability bias may still exist in our study and should be improved in future studies. Finally, it should be noted that our entire findings are based on the assumption that an individual will make rational decisions related to EMR access.

## Conclusions

Prior IS security research which adopted deterrence theory as its foundation has found that deterrence and detection practices can serve to regulate employees’ compliance intentions. While these findings are important, we argue that the literature can benefit from identifying the moderating effect of detection practices which are presented. To that end, we proposed and empirically validated a research model that drew from the impact of sanction severity and sanction certainty on hospital employees’ compliance intention. Further, the moderating influence of computer monitoring on above relationships is also investigated. Our findings revealed that both sanction severity and sanction certainty affect hospital employees’ compliance intention of the EMR privacy policy. More importantly, we found computer monitoring lowers the relationship between sanction certainty and compliance intention. By focusing on the moderating impact of computer monitoring, knowledge of deterrence theory is able to be augmented and diversified. Further, healthcare facilities can better secure the privacy quotient of EMR by adopting deterrence practices in conjunction with detection practices which should be carefully implemented to lower unexpected influences leading to possible breaches.

## Data Availability

The anonymous datasets from the present study are available from the corresponding author on reasonable request. No identifying/confidential patient data was collected.

## Appendix A

**Table A1:** Selected literature of deterrence theory in information security context

Studies	Exogenous variable	Endogenous variable	Dependent variable
Straub [13]	Deterrent certainty, deterrent severity		Computer abuse
Kankanhalli et al. [42]	Deterrent efforts, deterrent severity, preventive efforts		IS security effectiveness
Lee et al. [43]	Security policy, security awareness, security systems	Self-defense intention	Abuse by invaders/insiders
Pahnila et al. [36]	Sanctions	Intention to comply with IS security policy	Actual compliance of IS security policy
Herath & Rao [15]	Severity of penalty, Certainty of detection		Policy compliance intention
Herath & Rao [16]	Punishment severity, deterrent certainty, security policy attitude		SPCI
D’Arcy et al. [14]	Security policy, security education, training, and awareness program, computer monitoring	Perceived certainty of sanction, Perceived severity of sanction	IS misuse intention
D’Arcy & Hovav [39]	Security policy, security education, training, and awareness program, computer monitoring		IS misuse intention
Li et al. [41]	Detection probability, Sanction severity		Internet use policy compliance intention
Siponen et al. [38]	Deterrence		Actual compliance of information security policy
Hu et al. [37]	Perceived certainty of sanction, perceived severity of sanction, perceived celerity of sanctions		Intention to commit violation
Siponen & Vance [46]	Formal sanction, Informal sanction		Intention to violate IS security policy
Xue et al. [56]	Actual punishment	Punishment expectancy, perceived justice of punishment	Compliance intention
Guo et al. [57]	Attitude toward security policy, perceived sanction, perceived deterrent certainty	Attitude toward non-malicious security violation	Non-malicious security violation intention
Son [58]	Perceived deterrent certainty, perceived deterrent severity		Compliance of IS security policy
Hovav & D’Arcy [17]	Procedural countermeasure, technical countermeasure	Perceived certainty of sanction, perceived severity of sanction, moral belief	IS misuse intention
Guo & Yuan [19]	Organizational sanction, workgroup sanction	Personal self-sanction	Intention of information security violation
D’Arcy & Devarja [59]	Certainty*severity		Technology misuse intention
Chen et al. [60]	Punishment, certainty of control		Intention to comply with IS security policy
Cheng et al. [61]	Perceived certainty, perceived severity		IS security policy violation intention

Note:

1. An exogenous variable denotes a variable that is not caused by another variable in the model

2. An endogenous variable means a variable that is caused by one or more variable in the model

**Table A2:** Questionnaire

Constructs	Items	Source
Sanction severity	My hospital disciplines employees who break EMR privacy rules	Herath & Rao [15]
	My hospital terminates employees who repeatedly break EMR privacy rules	
Sanction certainty	If I don’t follow EMR privacy policies, I will be penalized	Siponen & Vance [46] Siponen et al. [38]
	I would be formally sanctioned if management learned that I had violated EMR privacy policy	
	I would be formally reprimanded if management learned that I had violated EMR privacy policy	
Computer monitoring	I believe that my hospital monitors any modification or altering of EMR by employees	D’Arcy et al. [14]
	I believe that my hospital monitors EMR usage activities to ensure that employees are performing only explicitly authorized tasks	
	I believes that my hospital reviews logs of employees’ EMR usage activities on a regular basis	
Intention to comply with EMR privacy policy	I intend to continue complying with EMR privacy policy in the future.	Venkatesh et al. [47]
	I will always try to comply with EMR privacy policy in my daily life.	
	I plan to continue to adhere with EMR privacy policy frequently.	

## Abbreviations

CM: Computer monitoring
EMR: Electronic medical records
ITC: Intention to comply
KMO: Kaiser-Meyer-Olkin
PCA: Principal component analysis
SC: Sanction certainty
SS: Sanction severity
